# Improving the Accuracy and Precision of Disease Identification When Utilizing Ehr Data for Research: the Case for Hepatocellular Carcinoma

**DOI:** 10.21203/rs.3.rs-4993106/v1

**Published:** 2024-10-18

**Authors:** Carrie R. Wong, Yvonne N. Flores, Analissa Avila, Lina Tieu, Catherine M. Crespi, Folasade P. May, Douglas Bell, Beth Glenn, Roshan Bastani

**Affiliations:** Vatche and Tamar Manoukian Division of Digestive Diseases, Department of Medicine, University of California, Los Angeles; UCLA Center for Cancer Prevention and Control and UCLA-Kaiser Permanente Center for Health Equity; UCLA Center for Cancer Prevention and Control and UCLA-Kaiser Permanente Center for Health Equity; UCLA Center for Cancer Prevention and Control and UCLA-Kaiser Permanente Center for Health Equity; UCLA Center for Cancer Prevention and Control and UCLA-Kaiser Permanente Center for Health Equity; Vatche and Tamar Manoukian Division of Digestive Diseases, Department of Medicine, University of California, Los Angeles; Division of General Internal Medicine, Department of Medicine, University of California, Los Angeles; UCLA Center for Cancer Prevention and Control and UCLA-Kaiser Permanente Center for Health Equity; UCLA Center for Cancer Prevention and Control and UCLA-Kaiser Permanente Center for Health Equity

## Abstract

**Objective:**

We assessed the performance of ICD codes to identify patients with hepatocellular carcinoma (HCC) in a large academic health system and determined whether employing an algorithm using a combination of ICD codes could deliver higher accuracy and precision than single ICD codes in identifying HCC cases using electronic health record (EHR) data.

**Results:**

The use of a single ICD code entry for HCC (ICD-9-CM 155.0 or ICD-10-CM C22.0) in our cohort of 1,007 established ambulatory care patients with potential HCC yielded 58% false positives (not true HCC cases) based on chart reviews. We developed an ICD code-based algorithm that prioritized positive predictive value (PPV), F-score, and accuracy to minimize false positives and negatives. The highest performing algorithm required at least 10 ICD code entries for HCC and the sum of ICD code entries for HCC to exceed the sum of ICD code entries for non-HCC malignancies. The algorithm demonstrated high performance (PPV 97.4%, F-score 0.92, accuracy 94%), which was internally validated (PPV 92.3%, F-score 0.90, accuracy 91%) using a separate sample of potential HCC cases. Our findings support the need to assess the accuracy and precision of ICD codes before using EHR data to study HCC more broadly.

## Introduction

Hepatocellular carcinoma (HCC) is the most common type of liver cancer among adults and the sixth leading cause of cancer deaths in the United States(1). Epidemiological trends for HCC vary based on race/ethnicity, sex, and age, and outcomes are conditional on patient and tumor characteristics and treatment options. Population-based estimates of the epidemiology and outcomes of HCC have been largely derived from Surveillance Epidemiology and End Results (SEER) or Veterans Affair (VA) administrative data(2, 3). SEER captures large patient samples using validated measures from histology and radiology reports but lacks treatment-related factors, which limits its ability to compare treatments and outcomes(4). While the use of a single International Classification of Diseases (ICD) code for HCC has been previously validated in the VA with a high positive predictive value (PPV) of 86%, findings from VA administrative data have limited generalizability beyond the veteran population(5).

Large data from health systems that provide longitudinal care for patients with HCC are needed to obtain more precise estimates of HCC epidemiology and treatment outcomes. A study based in an academic health system examined the accuracy of ICD, Ninth Revision, Clinical Modification (ICD-9-CM) codes from an administrative database to identify patients with HCC and found that a combination of two occurrences of ICD-9-CM codes for HCC and two ICD-9-CM codes for chronic liver disease and/or cirrhosis were needed to achieve an 87% PPV in capturing true HCC cases.(6) Since transitioning to the ICD, Tenth Revision, Clinical Modification (ICD-10-CM) coding system, the precision, accuracy, and validity of ICD codes in identifying HCC cases have not been thoroughly assessed using health system administrative data outside of SEER and VA sources.

We sought to assess the performance of ICD-9-CM and ICD-10-CM diagnostic codes to identify patients with HCC with established ambulatory care at a large academic health system and determine whether employing an ICD code-based algorithm could deliver higher accuracy and precision in identifying HCC cases.

## Methods

**Figure 1:**
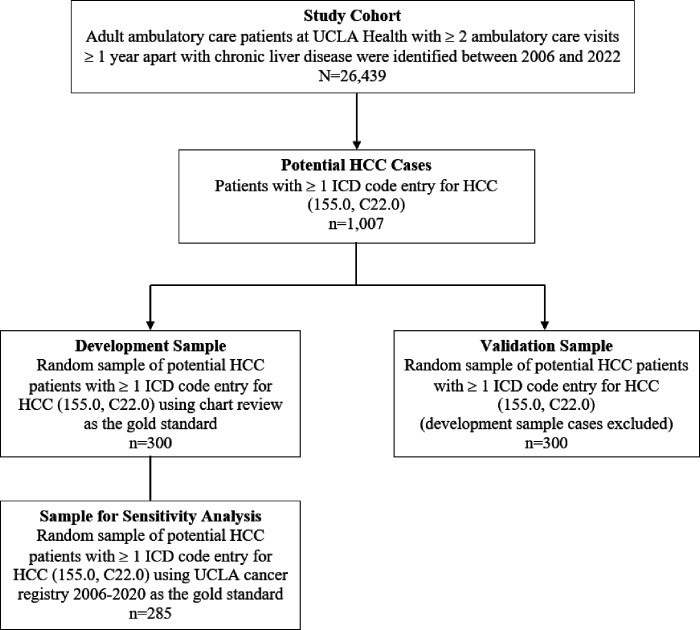
Flowchart of Study Population

We used ICD-9-CM and ICD-10-CM codes (Table 1 in Supplement) to assemble a study cohort of adult patients with chronic liver disease between 2006 and 2022 using electronic health record (EHR) data at an academic health system (UCLA Health), which included two hospitals and over 200 medical clinics across Southern California (Figure 1). All patients had a minimum of two ambulatory care visits in primary care at least one year apart to be considered established ambulatory care patients in the health system (n=26,439). We defined the follow-up period as the time between the first ICD code entry for HCC to the most recent encounter. We first queried for patients with potential HCC, which we defined as having at least one entry of ICD-9-CM (155.0) or ICD-10-CM (C22.0) code for HCC (n=1,007). To assess the performance of a single ICD code entry for HCC, we assembled a development sample, which was a random pool of 300 patients from the potential HCC sample, for chart review by three physicians (SJS, AB, SB) using a structured abstraction form. Separate chart reviews were conducted by a transplant hepatologist (CRW) for any discrepancies. HCC diagnosis was established using a combination of imaging, histology, and clinical notes in the EHR. Abstraction of clinical data from chart reviews provided information about true HCC cases (true positive) and false HCC cases (false positive), which served as the gold standard comparison. From our chart reviews using the development sample, we identified the most frequent non-HCC malignancies, which were included in our algorithm. Since the algorithm was based on the development sample using manual chart reviews as the gold standard, we also performed a sensitivity analysis using the institution’s cancer registry as a reference. This sensitivity analysis was applied to 285 patients from the development sample diagnosed with HCC between 2006 and 2020. The sensitivity analysis excluded 15 patients because they were diagnosed with HCC outside of the registry date range (e.g. after 2020 when registry data was unavailable at the time of this study).

Performance measurements, including sensitivity, specificity, PPV (precision), negative predictive value (NPV), F-score (harmonic mean of PPV and sensitivity), and accuracy (percentage of patients who were correctly classified) accompanied each algorithm iteration (Table 1). We selected the best performing algorithm with the highest PPV, F-score, and accuracy to reduce the number of false positive and negative cases. We internally validated the highest performing algorithm using a different random sample of 300 patients from the pool of potential patients with HCC (Figure 1).

Performance measurements of each algorithm were obtained using the Development Sample, which was a random pool of 300 potential HCC patients with at least 1 ICD code entry for HCC.

Algorithm 1 included an increasing number of ICD code entries for HCC using ICD codes 155.0 and C22.0. Performance measurements in Algorithm 2 were for exclusions of each respective non-HCC malignancy: secondary malignancy (ICD codes 197.7, C78.7), cholangiocarcinoma (ICD codes 155.1, C22.1), pancreatic cancer (ICD codes 157.9, C25.9), colorectal cancer (ICD codes 153.9, C18.9), or neuroendocrine tumor (ICD codes 209, 209.0, 209.00–209.03, 209.1, 209.10–209.17, 209.72, C7A.1, C7A.010-C7A.012, C7A.019-C7A.026, C7A.029, C7B.02). Algorithm 3 included the best performing iteration in Algorithm 1, which included at least 10 ICD code entries for HCC, and excluded each respective non-HCC malignancy, which were evaluated in Algorithm 2 (secondary malignancy, cholangiocarcinoma, pancreatic cancer, colorectal cancer, neuroendocrine tumor). Algorithm 4 included iterations from Algorithm 1, which included an increasing number of ICD code entries for HCC and required the sum of ICD code entries for HCC to exceed the sum of ICD code entries for non-HCC malignancies (secondary malignancy, cholangiocarcinoma, pancreatic cancer, colorectal cancer, neuroendocrine tumor). Highlighted are the highest performing iterations in Algorithm 1 and Algorithm 4.

## Results

The cohort of 26,439 established patients with ICD codes indicative of chronic liver disease included 1,007 patients with potential HCC based on a single ICD-9-CM or ICD-10-CM code entry. Chart reviews of the random sample of 300 potential HCC cases revealed that 58% of the potential cases of HCC were false positives (not true HCC cases). Of the 174 false HCC cases, 64 patients (36.8%) were found to have non-HCC malignancies, including 20 with cholangiocarcinoma, 16 with metastatic colorectal cancer, 9 with metastatic neuroendocrine tumors, 4 with metastatic pancreatic adenocarcinoma, and 15 with other metastatic malignancies. The second largest group of false positives (32.1%) included patients undergoing surveillance for HCC in the setting of chronic liver disease (n = 32) and other malignancies (n = 24) including history of renal cell carcinoma, breast cancer, and head and neck cancers. Other false HCC cases included patients who were found to have non-HCC liver lesions (n = 32; 18.4%) such as hemangiomas and hepatocellular adenomas or had diagnostics performed for other indications including abnormal liver enzymes (n = 22; 12.6%).

In the development sample (n = 300), the median age (interquartile range [IQR]) was similar between the true and false HCC cases, which were 64 (57–71) and 65 (53–73) years, respectively (p = 0.69). Median follow-up was also not significantly different between the HCC cases and non-cases: 6.4 (2.6–10.3) versus 5.7 (2.2–9.4) years, respectively (p = 0.21). Notably, there were significantly more females among false HCC cases (50%) compared to true HCC cases (31%), p < 0.01.

We first examined the frequency of entries for HCC using ICD codes based on prior work(6), which revealed that the best iteration of Algorithm 1 included at least 10 ICD code entries for HCC (Table 1). We then compared the performance metrics after excluding specific non-HCC malignancies that were frequently miscoded using ICD codes for HCC (Algorithm 2). We combined the best performing iteration from Algorithm 1 with exclusion of each non-HCC malignancy (Algorithm 3). To account for all common non-HCC malignancies, we developed Algorithm 4, which included the best performing iteration from Algorithm 1 and required the sum of ICD code entries for HCC to exceed the sum of ICD code entries for the most common non-HCC malignancies, including cholangiocarcinoma, colorectal cancer, pancreatic cancer, neuroendocrine tumor, and other secondary malignancies.

The best performing algorithm was an iteration of Algorithm 4 which had a of PPV of 97.4%, F-score of 0.92, and accuracy of 94% using the development sample. When we tested the algorithm using the cancer registry as the gold standard, the algorithm had similar performance metrics (PPV 94.3%, F-score 0.93, accuracy 94.4%). Our internal validation results revealed a consistently high performance with a PPV of 92.3%, F-score of 0.90, and accuracy of 91%.

## Discussion

This study assessed the performance of multiple ICD-9-CM and ICD-10-CM for HCC to precisely and accurately identify patients with HCC and longitudinal ambulatory care in a large academic health system. In contrast to findings from the VA Corporate Data Warehouse(3), a single ICD code for HCC performed poorly with a 58% false positive rate (42% accuracy). An algorithm requiring at least 10 ICD code entries for HCC in combination with the sum of HCC ICD code entries exceeding the sum of non-HCC malignancy ICD code entries identified true HCC cases with at least 90% accuracy and precision in our development and validation samples.

The discrepancy between the performance metrics of a single ICD code entry in our health system compared to the VA may be related to different coding practices and different patient populations including a higher prevalence of patients undergoing specialized cancer treatments in our health system. Compared to the VA, our health system includes patients with more female-specific malignancies, such as breast cancer, which require monitoring for liver metastasis. Specialized cancer treatments offered in our health system also require frequent surveillance imaging. As a referral center for specialty care, including transplant hepatology and hepatobiliary surgery, our health system likely encounters more consultations for evaluation of suspicious liver lesions, which may be miscoded using ICD-9-CM and ICD-10-CM codes for HCC. The PPV of the algorithm increased with an increasing number of ICD code entries for HCC as similarly reported in a previous study conducted in an academic health system(6).

### Limitations and Strengths

Several limitations should be considered when interpreting our findings. First, UCLA Health includes a tertiary care center, which receives referrals for specialized care in oncology, hepatobiliary surgery, and liver transplantation. Therefore, the prevalence of HCC is expectedly high in the study’s population, which could have inflated the PPV of our algorithm. Nonetheless, a higher prevalence of non-HCC malignancies would also be expected, which was countered by including the sum of ICD code entries for HCC to exceed the sum of ICD code entries for non-HCC malignancies in the algorithm. We also included additional performance metrics, such as the specificity and F-score, which are not as affected by disease prevalence. Second, while chart reviews were considered the gold standard to develop the best performing algorithm, human error during manual abstractions of EHR data could bias results. Third, the study cohort was defined using ICD codes for chronic liver diseases, which were similarly subject to misclassification and could have led to downstream effects on the development and performance of our algorithm. In recognition of these limitations, we tested the performance of the algorithm using cancer registry data, which identified HCC cases independently from our study team using separate criteria, as a sensitivity test which showed similar results.

## Conclusion

In conclusion, this study demonstrated that a combination of ICD-9-CM and ICD-10-CM codes for HCC can be used via an algorithm with over 90% accuracy and precision, whereas the use of a single ICD code entry to identify HCC cases requires caution in similar large health systems. With emerging new treatments for HCC(7) and use of artificial intelligence to capture HCC characteristics and outcomes using EHR data(8), large samples of patients with accurate, precise, and valid HCC diagnoses are needed to derive population-based estimates of treatment receipt and response, compare effectiveness of different treatments, and assess for differences in treatment uptake and outcomes by patient characteristics. Future studies that leverage EHR-based data to conduct studies about HCC epidemiology and treatment outcomes are encouraged to validate the accuracy and precision of ICD codes for HCC diagnoses before generating population-based estimates.

## Figures and Tables

**Table 1.** Performance measurements of ICD code-based algorithm iterations

	Sensitivity	Specificity	PPV	NPV	F-Score	Accuracy
**Algorithm 1: Increasing number of ICD code entries for HCC**						
^3^ 9 ICD code entries for HCC	88.1	93.7	91.0	91.6	0.90	91.3
^3^ 10 ICD code entries for HCC	88.1	94.8	92.5	91.7	0.90	92.0
^3^ 11 ICD code entries for HCC	85.7	94.8	92.3	90.2	0.89	91.0
**Algorithm 2: Exclusion of each non-HCC malignancy with ^3^ 1 ICD code entry for HCC**						
Secondary malignancy	95.2	17.8	45.6	83.8	0.62	50.3
Cholangiocarcinoma	89.7	17.2	44.0	69.8	0.59	47.7
Pancreatic cancer	100	0	42.0	0	0.59	42.0
Colorectal cancer	100	0	42.0	0	0.59	42.0
Neuroendocrine tumor	100	0	42.0	0	0.59	42.0
**Algorithm 3: ^3^ 10 ICD code entries for HCC and exclusion of each non-HCC malignancy**						
Secondary malignancy	83.3	95.4	92.9	88.8	0.88	90.3
Cholangiocarcinoma	77.8	98.9	98.0	86.0	0.87	90.0
Pancreatic cancer	88.1	94.8	92.5	91.7	0.90	92.0
Colorectal cancer	88.1	94.8	92.5	91.7	0.90	92.0
Neuroendocrine tumor	88.1	94.8	92.5	91.7	0.90	92.0
**Algorithm 4: Increasing number of ICD code entries for HCC and sum of ICD code entries for HCC exceed sum of ICD code entries for non-HCC malignancies**						
^3^ 9 ICD code entries for HCC	88.1	97.1	95.7	91.8	0.92	93.3
^3^ 10 ICD code entries for HCC	88.1	98.3	97.4	91.9	0.92	94.0
^3^ 11 ICD code entries for HCC	85.7	98.3	97.3	90.5	0.91	93.0

PPV; positive predictive value, NPV; negative predictive value; HCC, hepatocellular carcinoma

## Data Availability

Study data cannot be openly shared to protect the private health information of patients at UCLA Health.

## Abbreviations

HCC: hepatocellular carcinoma
SEER: Surveillance Epidemiology and End Results
VA: Veterans Affair
ICD: International Classification of Diseases
PPV: positive predictive value
ICD-9-CM: ICD, Ninth Revision, Clinical Modification
ICD-10-CM: ICD, Tenth Revision, Clinical Modification
NPV: negative predictive value
EHR: electronic health records
